# How Different Are the Molecular Mechanisms of Nodal and Distant Metastasis in Luminal A Breast Cancer?

**DOI:** 10.3390/cancers12092638

**Published:** 2020-09-16

**Authors:** Petr Lapcik, Anna Pospisilova, Lucia Janacova, Peter Grell, Pavel Fabian, Pavel Bouchal

**Affiliations:** 1 Department of Biochemistry, Faculty of Science, Masaryk University, 62500 Brno, Czech Republic; 409180@mail.muni.cz (P.L.); 437235@mail.muni.cz (A.P.); 379939@mail.muni.cz (L.J.); 2 Department of Comprehensive Cancer Care, Masaryk Memorial Cancer Institute, 65653 Brno, Czech Republic; grell@mou.cz; 3 Department of Oncological Pathology, Masaryk Memorial Cancer Institute, 65653 Brno, Czech Republic; fabian@mou.cz

**Keywords:** breast cancer, lymph node, distant metastasis, GSEA, pathway, inhibitor

## Abstract

**Simple Summary:**

Lymph node status is one of the best prognostic factors in breast cancer, however, its association with distant metastasis is not straightforward. Here we compare molecular mechanisms of nodal and distant metastasis in molecular subtypes of breast cancer, with major focus on luminal A patients. Our results indicate that lymph node positivity is associated with NF-κB and Src pathways and is related to high risk of distant metastasis in luminal A patients. Distant metastasis of lymph node negative tumors is related to cell proliferation control and thrombolysis, whereas distant metastasis of lymph node positive tumors is associated mostly to immune response. These mechanisms vary in other molecular subtypes. Our data indicate that the management of breast cancer and prevention of distant metastasis requires stratified approach based on targeted strategies.

**Abstract:**

Lymph node status is one of the best prognostic factors in breast cancer, however, its association with distant metastasis is not straightforward. Here we compare molecular mechanisms of nodal and distant metastasis in molecular subtypes of breast cancer, with major focus on luminal A patients. We analyze a new cohort of 706 patients (MMCI_706) as well as an independent cohort of 836 primary tumors with full gene expression information (SUPERTAM_HGU133A). We evaluate the risk of distant metastasis, analyze targetable molecular mechanisms in Gene Set Enrichment Analysis and identify relevant inhibitors. Lymph node positivity is generally associated with NF-κB and Src pathways and is related to high risk (OR: 5.062 and 2.401 in MMCI_706 and SUPERTAM_HGU133A, respectively, *p* < 0.05) of distant metastasis in luminal A patients. However, a part (≤15%) of lymph node negative tumors at the diagnosis develop the distant metastasis which is related to cell proliferation control and thrombolysis. Distant metastasis of lymph node positive patients is mostly associated with immune response. These pro-metastatic mechanisms further vary in other molecular subtypes. Our data indicate that the management of breast cancer and prevention of distant metastasis requires stratified approach based on targeted strategies.

## 1. Introduction

Breast cancer is the most often diagnosed cancer, and the most lethal cancer in women worldwide in absolute numbers [1]. In most cases, primary tumors do not represent the main cause of the death, and about 90% of deaths is caused by development of the secondary tumors, metastases [2]. The presence of cancer cells in regional lymph nodes is an established prognostic factor for development of distant metastases and patient survival [3,4,5,6,7,8]: regional lymph node metastases have been observed in about one third of breast cancer patients at the time of diagnosis [9], and patients with more than three positive axillary lymph nodes have been shown to have five-fold higher probability of distant metastasis development [10,11,12,13]. The relationship between lymph node and distant metastasis is, however, not straightforward in two typical cases: (i) approximately one third of breast cancer patients with negative regional lymph nodes develop distant metastasis in disagreement with the prognosis based on lymph node status [14,15,16], and (ii) one third of breast cancer patients with positive lymph nodes do not develop distant metastases at all [17,18]. This leads to underestimation of the risk in the first case, and overtreatment in the second case in the clinical practice. As such, the mechanisms that develop lymph node metastases are hypothetically at least partially, or even completely different from the mechanism responsible for distant metastasis since cancer cells spreading to lymph nodes utilize transport modes different from the cells that spread via blood circulation [19].

A confounding factor that contributes to the unstraightforward association between lymph node and distant metastasis is the molecular heterogeneity of breast cancer. It is well known that different combinations of estrogen receptor (ER), progesterone receptor (PgR), human epidermal growth factor receptor 2 (Her2) status [20] and tumor grade define at least four molecular subtypes of breast cancer, luminal A, luminal B, Her2-enriched (Her2+), and basal/triple-negative [21]. Patients of luminal A and basal subtype form the regional lymph node metastases less frequently [10,22,23,24,25,26,27,28,29] (see Table S1 for overview of the studies) and non-luminal tumors tend to metastasize into distant organs more often than luminal tumors [22,27,29,30] (see Table S2 for overview of the studies). Although the conclusions made in these individual studies correspond to their different experimental design, the results indicate that the uncertain association between lymph node and distant metastasis is further complicated by molecular heterogeneity of breast cancer.

Buonomo et al. compared lymph node involvement and occurrence of distant metastases in 324 breast cancer patients and indicated that Her2+ patients and the basal patients show the highest and the lowest risk, respectively, of distant metastasis based on lymph node status [31]. The association between lymph node and distant metastasis in non-luminal patients was overall stronger than in luminal patients. Within the luminal patient group, luminal A patients showed lower chance on distant metastasis in lymph node positive cases than patients of luminal B subtype. As there was no independent study specifically comparing the association between lymph node and distant metastasis in luminal A subtype, we have analyzed a newly established cohort of 706 patients treated at Masaryk Memorial Cancer Institute (MMCI) and used it to confirm whether the risk of distant metastasis is associated with nodal positivity of luminal A patients. To validate these findings in an independent cohort and to study the underlying molecular mechanisms of lymph node and distant metastasis, we analyzed the dataset SUPERTAM_HGU133A [32] that was available, as the only one, for immediate download including the information on lymph node and distant metastasis and the gene expression microarray profiles. Using the data from the second dataset, we identified molecular pathways enriched in tumors developing distant metastasis in relation to their lymph node status at the time of diagnosis. Based on the literature research of the enriched pathways, corresponding key biomarkers and their validation data referenced here, we then proposed a panel of currently available inhibitors targeting these pathways and discuss their potential applicability for improvement of breast cancer treatment.

## 2. Results

### 2.1. Association between Lymph Node Status and Development of Distant Metastasis in Two Independent Patient Cohorts

To study the association between lymph node status and development of distant metastasis, we enrolled a new cohort of 706 patients initially diagnosed at MMCI between 2004 and 2007 with at least 5-year follow-up, referred as MMCI_706, containing 381 luminal A, 218 luminal B, 32 Her2+ and 75 basal patients, please see Table S3 for descriptive statistics of lymph node positive (N1) “cases” and lymph node negative (N0) “controls” at the time of diagnosis. Then we evaluated the risk of distant metastasis development in N1 vs. N0 patients in all breast cancer subtypes. In Table 1 we show the highest risk (the highest odds ratios, *p* < 0.05) of distant metastasis related to lymph node status for luminal A subtype, followed by basal and luminal B. In order to validate these results in an independent patient cohort containing full gene expression data for subsequent pathway analyses, we analyzed SUPERTAM_HGU133A dataset of 836 patients containing 341 luminal A, 281 luminal B, 71 Her2+ and 143 basal patients with Affymetrix Human Genome U133A gene expression data and performed the same evaluation as for MMCI_706 dataset. We found the highest risk of distant metastasis related to lymph node status for luminal A subtype (Table 1). The data from both datasets show that luminal A tumors (OR 5.062 and 2.401, *p* < 0.05) have the strongest association between lymph node metastasis and distant metastasis.

### 2.2. Molecular Mechanisms of Metastatic Events Depend on Breast Cancer Subtype

To understand the molecular basis of lymph node vs. distant metastasis in breast cancer subtypes, we compared gene expression in N1 vs. N0 primary tumors at the time of diagnosis and in distant metastasis positive (M1) and distant metastasis negative (M0) primary tumors during the follow-up for all breast cancer subtypes in SUPERTAM_HGU133A dataset. Then we performed GSEA analyses of the gene expression data for all subtypes separately to identify pathways associated with lymph node and distant metastasis.

**Figure 1:**
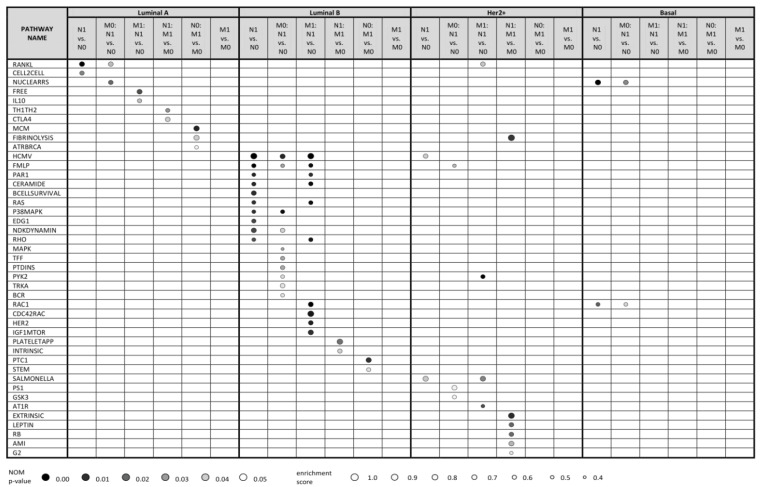
Top 10 statistically significant pathways (NOM *p*-value < 0.05) resulting from Gene Set Enrichment Analysis (GSEA) based on transcriptomic profiles of breast cancer patients from SUPERTAM_HGU133A dataset. Using SCMOD2 classifier, patients were classified into 4 breast cancer molecular subtypes, resulting in 341 luminal A, 281 luminal B, 71 Her2+ and 143 basal patients. To demonstrate differences in molecular mechanisms included in lymph node metastasis (N1) and distant metastasis (M1), expression profiles were compared under 6 conditions (N1 vs. N0, M0: N1 vs. N0, M1: N1 vs. N0, N1: M1 vs. M0, N0: M1 vs. M0, M1 vs. M0) related to these two events. Pathways were searched against Biocarta pathway database. Up to 10 statistically significant pathways are shown for each condition within each subtype. No statistically significant pathways were identified under M1 vs. M0 condition for patients with all breast cancer subtypes. M0: N1 vs. N0, lymph node positive vs. negative tumors; all distant metastasis negative; M1: N1 vs. N0; lymph node positive vs. negative tumors; all distant metastasis positive; M1 vs. M0, distant metastasis positive vs. negative tumors; N0: M1 vs. M0, distant metastasis positive vs. negative tumors; all lymph node negative; N1: M1 vs. M0; distant metastasis positive vs. negative tumors; all lymph node positive; N1 vs. N0, lymph node positive vs. negative tumors.

Figure 1 shows up to 10 top enriched pathways for all kinds of comparisons with nominal (NOM) *p*-value < 0.05 (see Table S6 for full data). The data clearly show that the molecular pathways enriched in lymph node positive (vs. negative) primary tumors, distant metastasis positive (vs. negative) tumors and relevant combinations thereof are highly distinct for every subtype. This is further detailed below.

#### 2.2.1. Luminal A Subtype

GSEA analysis of gene expression profiles of primary luminal A tumors showed enrichment of two pathways, RANKL and CELL2CELL, in comparison of lymph node positive vs. negative tumors (N1 vs. N0 in Figure 1). These pathways participate in NF-κB activation and in cell adhesion and migration, respectively, and are thus biologically relevant for the local invasion. RANKL pathway was enriched also in lymph node positive vs. negative luminal A tumors in a subgroup of distant metastasis negative tumors (M0: N1 vs. N0 comparison), together with NUCLEARRS pathway. Similarly, FREE and IL10 pathways (both involving pro-inflammatory cytokines connected to NF-κB pathway) were enriched in lymph node positive vs. negative tumors in a distant metastasis positive tumor subgroup (M1: N1 vs. N0 comparison). Thus, we suggest that NF-κB pathway plays an important role in migration of cancer cells in regional lymph nodes in luminal A patients regardless of distant metastatic events.

On the other hand, GSEA analysis of primary luminal A tumors showed no enriched pathways in comparison between distant metastasis positive vs. negative tumors (M1 vs. M0). Interestingly, comparisons of distant metastasis positive vs. negative tumors within the subset of lymph node negative tumors (N0: M1 vs. M0) and distant metastasis positive vs. negative tumors within the subset of lymph node positive tumors (N1: M1 vs. M0) led to enrichment of different sets of pathways. Especially, N0: M1 vs. M0 comparison indicated enrichment of MCM, ATRBRCA and FIBRINOLYSIS pathways consisting of proteins participating in cell cycle progression, DNA repair and plasminogen activation, respectively. On the other hand, N1: M1 vs. M0 comparison resulted in enrichment of TH1TH2 and CTLA4 pathways that involve proteins of specific immune response. This indicates distinct mechanisms of distant metastasis in lymph node positive and lymph node negative patients.

Comparisons of expression profiles between N1 and N0 or M1 and M0 patients within each condition are visualized using heatmap and hierarchical cluster analysis that show more conserved profiles in N1 than in M1 patients, probably reflecting different organs affected by distant metastasis (Figure S1). Volcano plots show higher number of differentially expressed genes in lymph node positive to lymph node negative comparisons than in distant metastasis positive to distant metastasis negative comparisons (Figures S2–S7).

**Figure 2:**
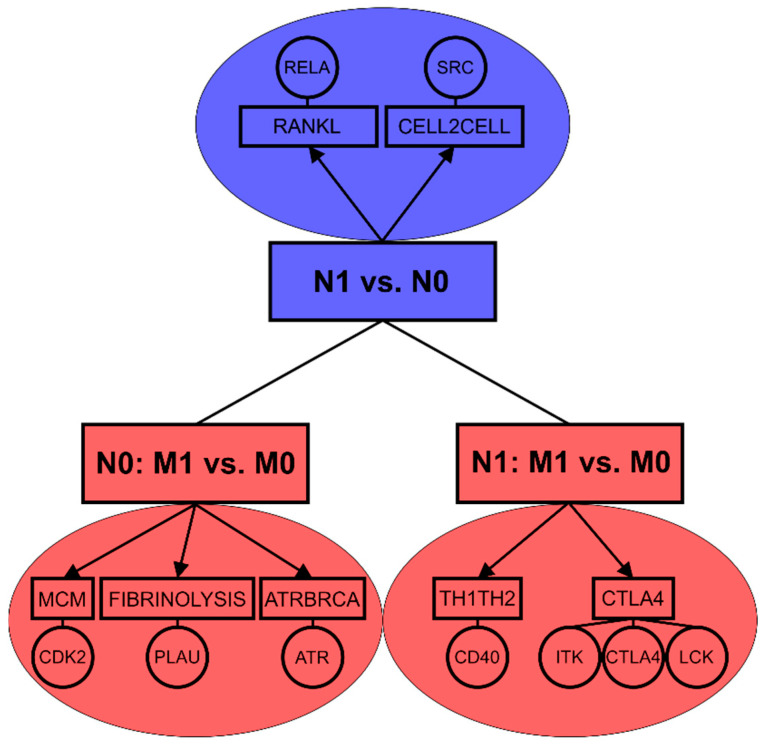
Enriched pathways and key biomarkers associated with lymph node metastasis (N1 vs. N0) and distant metastasis based on lymph node status (N0: M1 vs. M0, N1: M1vs.M0) in luminal A breast tumors.

In a summary, while lymph node positivity of luminal A primary tumors seems to be associated with NF-κB pathway, the occurrence of distant metastasis appears to be connected with stronger mechanisms related to proliferation and cell cycle progression (Figure 2). The data confirm that molecular mechanisms of lymph node and distant metastasis are highly distinct even in luminal A subtype which exhibited the strongest association between lymph node metastasis and distant metastasis in both tested patient cohorts.

#### 2.2.2. Luminal B Subtype

GSEA analysis of gene expression profiles of luminal B tumors uncovered 31 pathways enriched in lymph node positive primary tumors (N1) in comparison with their lymph node negative (N0) counterparts (see Table S6 and Figure 1). Further, 14 and 31 pathways were statistically significantly enriched also in M0: N1 vs. N0; and M1: N1 vs. N0 comparisons, respectively. From these, CERAMIDE, RAS, P38MAPK and EDG1 pathways are involved in cell proliferation, FMLP and BCELLSURVIVAL pathways in immune response and RAS and RHO pathways in cytoskeletal rearrangement that could promote cell migration. Enriched pathways frequently included MAP kinase signaling cascades and phosphatidylinositol kinase activity. Similarly, as in luminal A breast cancer, some of these pathways (such as HCMV, FMLP, CERAMIDE, RAS) contain enriched proteins of NF-κB pathway.

Molecular changes associated with distant metastasis in luminal B patients were distinct in lymph node positive and lymph node negative patients, similarly as in luminal A tumors: lymph node negative tumors with developed distant metastases exhibited enrichment of PTC1 and STEM pathways representing Sonic Hedgehog signaling and cytokines supporting immune response, respectively. On the other hand, lymph node positive tumors that developed distant metastasis had enriched INTRINSIC and PLATELETAPP pathways that integrate the factors participating in coagulation cascade and platelet clotting, respectively.

These results indicate high number of deregulated pathways in primary luminal B tumors associated with lymph node metastasis. Moreover, pathways enriched in luminal B tumors with distant metastasis were distinct in lymph node positive and lymph node negative patients.

#### 2.2.3. Her2+ Subtype

GSEA analysis of gene expression profiles of Her2+ tumors uncovered SALMONELLA and HCMV pathways weakly enriched in lymph node positive primary tumors (N1) compared to their lymph node negative (N0) counterparts (Figure 1). These pathways participate in cytoskeletal remodeling. Moreover, HCMV pathway includes enriched MAP kinases and NF-κB transcription factor RELA. Similarly, FMLP, PS1, and GSK3 pathways were weakly enriched in M0: N1 vs. N0 comparison, contributing to immune response, Notch and Wnt signaling, NF-κB activation and beta-catenin. PYK2, RANKL, SALMONELLA and AT1R pathways were enriched in M1: N1 vs. N0 comparison, participating in cell cycle progression via MAP kinase cascade, NF-κB activation, cytoskeletal remodeling and c-Jun activation, respectively. In the case of distant metastasis, several pathways related to coagulation and cell cycle regulation were enriched in N1: M1 vs. M0 comparison, however, the number of cases in this comparison was rather low.

#### 2.2.4. Basal Subtype

Additionally, the number of enriched pathways associated with lymph node and distant metastasis was low in patients of basal subtype, primarily because of the low number of basal tumors available in SUPERTAM_HGU133 dataset. Only NUCLEARRS and RAC1 pathways affecting lipid and xenobiotic metabolism and cytoskeletal structure, respectively, were enriched in lymph node positive patients, as well as in their distant metastasis negative subset (M0: N1 vs. N0 comparison). No pathways were enriched in distant metastasis positive vs. negative basal tumors, possibly due to high heterogeneity in this subtype [33].

In a summary, our results clearly show that enriched pathways and underlying molecular mechanisms strongly differ between primary tumors that form lymph node vs. distant metastasis. In addition, patterns of enriched pathways related to nodal or distant metastasis are dependent on the molecular subtype of the breast cancer. Nevertheless, some components of these enriched pathways are shared among tumors of different subtypes, namely pleiotropic transcription factors of NF-κB family in tumors spreading through the lymphatic vessels or plasminogen-activating proteases in tumors disseminated via bloodstream.

### 2.3. Inhibitors of Pathways Enriched Specifically in Metastatic Luminal A Tumors

As shown above, the best associations between lymph node and distant metastasis were found for luminal A subtype. However, the pathways enriched in lymph node vs. distant metastatic tumors were highly distinct even in luminal A subtype. Although chemotherapy in adjuvant treatment generally decreases the risk of distant metastasis (Table S4), it is associated with number of undesirable effects for the patients. Development of more targeted therapies is of the clinical need and identification of pathways and targets responsible for various modes of metastasis is the key step toward. We selected luminal A as a model subtype and proposed a panel of potential anti-metastatic inhibitors based on literature search of GSEA analysis results. We identified a panel of total 42 inhibitors targeting all key pathways enriched in lymph node and distant metastasis of luminal A tumors: RANKL, CELL2CELL, TH1TH2, CTLA4, MCM, FIBRINOLYSIS and ATRBRCA BIOCARTA pathways, see Table 2. We comment on their role in breast cancer metastasis in Discussion. Table 3 summarizes results of their testing in clinical trials relevant for breast cancer treatment.

## 3. Discussion

### 3.1. Lymph Node and Distant Metastasis are Based on Different Molecular Mechanisms

Lymph node status is generally considered as one of the best prognostic factors in breast cancer [3]. However, our data from two patient cohorts, MMCI_706 and SUPERTAM_HGU133A, show ability of (not only) lymph node positive, but also lymph node negative tumors to develop distant metastases. Specifically, 20.1% and 36.9% of lymph node positive MMCI and SUPERTAM_HGU133A patients, respectively, developed distant metastasis during the follow-up period. This well corresponds with 18.5% in a relevant study by Colzani et al. [62] and 29.9% by Tchou et al. [63]. On the other hand, 6.5% and 25.1% of lymph node negative MMCI_706 and SUPERTAM_HGU133A patients, respectively, also developed distant metastases, which indicates that absence of lymph node dissemination at the time of diagnosis does not exclude development of distant metastasis in the follow-up period. These results correspond with 5.8% and 13.2% by Colzani et al. [62] and Tchou et al. [63], respectively, keeping in mind different populations and other confounding factors that may affect the statistics in the individual studies. This observation is also well supported by the fact that tumor cells spread via different ways to form lymph node, or distant metastasis—lymphatic vessels, or bloodstream, respectively. We thus hypothesized that molecular mechanisms responsible for nodal and distant metastasis are different.

Indeed, GSEA analysis confirmed this premise: lymph node metastasis was associated with number of pathways that include transcription factors of the NF-κB family which were previously associated with the lymph node metastasis of breast tumors [64]. On the other hand, distant metastasis was primarily connected to pathways regulating stronger mechanisms related to cell cycle and proliferation control, in agreement with Chowdhury et al. [65]. Other key pathways related to distant metastasis involved fibrinolytic proteases including urokinase plasminogen activator (uPA), well known in term of distant metastasis in breast cancer [66].

### 3.2. Molecular Mechanism Associated with Lymph Node and Distant Metastasis Are Also Dependent on Breast Cancer Subtype

Association between lymph node and distant metastasis is further affected by the molecular subtype in breast cancer and thus dependent on the hormonal background of the tumors. Buonomo et al. [31] revealed strong association between N1 and M1 status in patients of Her2+ subtype. We observed this connection for Her2+ in SUPERTAM_HGU133A dataset and showed the similar association for luminal A subtype in both tested cohorts. In term of molecular mechanisms, lymph node positive luminal A tumors exhibited enrichment of NF-κB related proteins as well as intercellular interaction pathway including Src proto-oncogene. Both NF-κB transcription factors and Src proto-oncogene were previously connected with tumorigenesis [67] and breast cancer metastasis [68]. Distant metastasis developed in lymph node negative luminal A patients was associated with cell cycle, DNA repair and immune response mechanisms [69,70,71]. In lymph node positive luminal A patients, distant metastasis was related to mechanisms of T lymphocyte differentiation and activation [72,73] (Figure 2).

Lymph node positive luminal B tumors were enriched in pathways related to cell proliferation, immune response and cytoskeletal changes such as enrichment of PIK3CA, MAP kinases, NF-κB factors and RHOA, pathways which play role in breast cancer progression [67,74,75,76]. Distant metastasis of luminal B tumors was associated with activation of Sonic Hedgehog proliferative signals and production of immunogenic cytokines in lymph node negative patients at the time of diagnosis, but with coagulation pathways in lymph node positive patients. These mechanisms were already associated with breast cancer progression and metastasis [77,78,79,80].

Occurrence of lymph node metastases in patients of basal subtype was associated with enrichment of proteins that participated in cytoskeletal reorganization and enhanced lipid and xenobiotic metabolism. Of these, peroxisome proliferator-activated receptor delta (PPARD) protein showed increased expression in breast cancer with negative impact on relapse free survival [81]. Expression of PPARD in breast cancer cells increased cell migration in vitro and induced creation of lung metastases in vivo [81]. ABCB1 and ABCC3 proteins, members of ATP binding cassette (ABC) transporter family, are associated with chemoresistance in breast cancer cells [82,83].

Based on the above findings, we assume unique pattern of molecular processes leading to nodal and distant metastases, which further differ in breast cancer subtypes. We selected luminal A subtype as a model to propose inhibitors for both metastatic modes based on the current literature to suggest potential improvements in the treatment.

### 3.3. Treatment of Luminal A Patients and Possibilities of Therapy Modulation

Luminal A patients from MMCI_706 and SUPERTAM_HGU133A cohorts exhibited the strongest association between lymph node positivity and distant metastasis development. Moreover, patients of luminal A subtype, despite the good prognosis and advances in therapy, comprise significant part of distant metastatic cases, 12.6% to 45.4% in various studies [22,27,29,30]. Current treatment of luminal A patients typically includes hormonal therapy [84] based on tamoxifen or aromatase inhibitors such as letrozole or exemestane [85]. Patients with high risk of tumor recurrence receive chemotherapy (typically based on anthracyclines and taxanes), however, these well differentiated tumors poorly respond to it. In case of metastatic luminal A patients, endocrine therapy and chemotherapy is administered. Other treatment possibilities include more targeted therapies—cyclin-dependent kinase 4 and 6 inhibitors (palbociclib, ribociclib, abemaciclib) that were recently approved by FDA in combination with hormone therapies for treatment of ER+ Her2- advanced breast cancer [86]. For example, palbociclib showed in combination with letrozole [87] and in combination with fulvestrant [88] increased progression-free survival (PFS) of hormonal receptor positive Her2- breast cancer patients compared to control patient groups. Another approved targeted treatment for metastatic breast cancer is mTOR inhibitor everolimus that in combination with exemestane demonstrated a 4.7-month improvement in PFS compared to exemestane alone [89].

Despite actual progress in breast cancer therapy and undergoing clinical studies, the treatment still requires further development to improve patient outcomes, because up to 13.6% of breast cancer patients (diagnosed in stage I–III) still develop bone metastasis within 15 years of follow-up [90]. We have proposed 42 inhibitors of potential therapeutic targets found using GSEA analysis (Table 2) that have previously exhibited potential to suppress luminal A breast cancer in vitro and eventually in vivo. To our knowledge, 16 of these compounds were clinically tested in patients with solid tumors (Table 3) and 11 of these were examined for clinical use in breast cancer patients (marked in Table 3), and some of these trials already showed beneficial activity of these compounds in breast cancer patients (please see Outcomes of clinical trials in Table 3). The corresponding molecular mechanisms and the relevant clinical scenarios are discussed in the following paragraphs.

### 3.4. NF-κB, Intercellular Adhesion and Nuclear Proteins are Potential Therapeutic Targets in Lymph Node Positive Luminal A Patients (N1 vs. N0)

Lymph node positive tumors compared to lymph node negative ones (N1 vs. N0) of luminal A subtype exhibited enrichment of RANKL and CELL2CELL pathways. Receptor activator of nuclear factor NF-κB (RANK) and its ligand (RANKL) are involved in progression of breast cancer [91,92]. Moreover, RANKL pathway is a downstream pathway in progesterone signalization [93]. In PgR-positive tumor cells, progesterone induces up-regulation of RANKL and enhances proliferation. Hence, we suggest that inhibition of RANKL could have significant anti-tumor effect in these tumors [93]. In GSEA results, NF-κB transcription factor RELA (p65) was enriched in RANKL pathway. RELA was overexpressed in lymph node positive vs. negative luminal A primary tumors in the set of 48 luminal A breast tumors at both protein and transcript level together with another NF-κB modulators (Table S5, [64]) that exhibited activating role on migration and invasion capacity of MCF7 breast cancer cells (Figure 3B in [94] and Figure 1C in [95]). Denosumab specifically binds to RANKL preventing NF-κB activation by RANKL cascade [96]. Denosumab already showed potential for clinical use in breast cancer treatment by reducing bone turnover and bone events in metastatic breast cancer patients in phase II trial [34]. Moreover, denosumab improved disease-free survival (DFS) in ER+, non-metastatic breast cancer patients by decreasing risk for osteoporosis and bone fractures (phase III) [35]. Although some studies showed that denosumab does not improve disease-related and survival outcomes for women with high-risk and metastatic breast cancer [97,98], its efficacy in these clinical trials was not assessed for specific breast cancer subtypes. Other compounds showed ability to inhibit NF-κB pathway in vitro [99,100,101,102]. From these inhibitors, curcumin was studied in phase I clinical trial in breast cancer patients [40]. Moreover, curcumin in combinatorial therapy reduced inflammation and pain in breast cancer patients with aromatase-induced musculoskeletal symptoms [41].

CELL2CELL pathway consists of proteins participating in intercellular interactions. These include proto-oncogene Src that was found to play an important role in metastatic spread of breast cancer cells to bones [68] and in resistance to anti-hormonal therapy [103,104]. Its expression was associated with axillary lymph node positivity in the set of 392 tamoxifen-treated ER-positive breast tumors (Table 2 in [105]) and its inhibition as well as siRNA knockdown led to reduced MCF7 cell migration and E-cadherin induction (Figures 1B, 2A,B and 3 in [106]). This supports Src association with metastatic potential. Src inhibitor dasatinib, FDA-approved compound for the treatment of chronic myeloid leukemia, was found to suppress resistance of breast cancer cells to endocrine therapy [107,108] and to doxorubicin [109]. Dasatinib in phase II clinical trials exhibited limited single-agent activity in ER+ patients [37] and also showed potential in combinatory therapies with zoledronic acid in ER+ patients [38] as well as with paclitaxel in metastatic breast cancer patients [36]. Moreover, dasatinib in combination with trastuzumab prolonged progression-free survival in Her2+ breast cancer patients [39]. Other Src inhibitors showed potential in vitro for treatment of ER+ breast cancer, namely PP2, [110], nobiletin [111] and (-)-Liriopein B [112].

Based on clinical trials of the selected NF-κB and Src inhibitors we presume the highest potential of denosumab and dasatinib for NF-κB and Src-targeted treatment of lymph node positive luminal A breast cancer patients.

### 3.5. Inhibition of DNA-Repair, Cell Cycle Control and Plasminogen-Activating Proteases Could be Beneficial for Treatment of Lymph Node Negative Luminal A Patients to Block Development of Distant Metastasis (N0: M1 vs. M0)

Lymph node negative luminal A tumors that developed distant metastases during follow-up period versus those that did not develop distant metastasis exhibited enrichment of MCM, FIBRINOLYSIS and ATRBRCA pathways. Of these, cyclin-dependent kinase 2 (CDK2) belonging to minichromosome maintenance protein complex (MCM) pathway plays an important role in cell cycle regulation [69]. CDK2 activity is a significant prognostic factor for relapse, especially in node-negative breast cancer, as confirmed in the set of 284 patients (Table 1 in [113]). siRNA knockdown of CDK2 retained MCF7 cells in G1 cell cycle phase (Figure 2B in [114]) and moreover, CDK2 inhibition slowed the proliferation of MCF7 cells down (Figure 3 in [114]). Expression of MCM2, MCM4 and MCM6 was associated with histological grade in the set of 3520 breast tumors (Figure 7 in [115]). Moreover, high expression of MCM2, MCM4 and MCM6 was related to shorter relapse-free survival in the set of 2069 luminal A breast cancers (Figure 11 in [115]). We have found total 22 compounds (Table 3) capable of inhibiting CDK2 in luminal A breast cancer models in vitro with inhibitory effects on cell viability and/or motility. These inhibitors include clinically tested alisertib [116], cepharanthine [117], roscovitine [118], norcantharidin [119], lycopene [120,121,122], troglitazone [123] and SNS-032 [124]. Alisertib displayed potential for treatment of endocrine-resistant, ER+ metastatic breast cancer patients in combination with fulvestrant (phase I clinical trial) [50] and a prolonged median PFS in patients with advanced breast cancer in combination with paclitaxel (phase II) [49]. Cepharantine decreased adjuvant chemotherapy-induced bone marrow suppression, leukopenia and thrombocytopenia in breast cancer patients [125]. However, the anti-tumor effect of cepharantine in breast cancer patients has not been studied. Roscovitine in combination with capecitabine underwent phase II clinical trial in metastatic breast cancer patients [53]. Roscovitine exhibited clinical potential in patients with nasopharyngeal carcinomas [55] and with other several advanced solid tumors [54]. Norcantharidin is clinically used drug for treatment of liver cancer in China [56]. Lycopene and troglitazone showed clinical potential for prostate cancer treatment [57,58], and SNS-032 was well tolerated in patients with advanced solid tumors in phase I clinical trial [59].

FIBRINOLYSIS pathway consists of proteases inducing coagulation cascade. Urokinase-type plasminogen activator (PLAU) is associated with increased risk of metastasis in breast cancer [70] and is considered as the strongest indicator of poor prognosis in patients with metastatic breast cancer (Figure 3 in [126]). Higher PLAU levels were also found as a strong predictor of locoregional and distant recurrence in the set of 1119 breast tumors (Table 4 in [127]). PLAU expression was significantly associated with a shorter distant metastasis-free survival (DMFS) (Figure 4A in [128]) and increased risk of distant metastasis (Table 2 in [128]). Nguyen et al. demonstrated that PLAU enhances MCF7 cell migration by uPAR-dependent mechanism (Figure 1 in [129]). Mesupron and nimbolide are PLAU inhibitors with potential for luminal A breast cancer treatment in vitro [130,131]. Moreover, mesupron increased PFS of Her2- metastatic breast cancer patients treated with capecitabine in phase II trial [60]. Mesupron also indicated potential for treatment of pancreatic cancer (phase II) [61].

ATRBRCA pathway includes factors mediating cell response to DNA damage, such as Ataxia Telangiectasia and Rad3 related (ATR) factor [71]. High ATR expression was associated with a poor survival in the set of 385 breast tumors (Figure 1C in [132]). ATR inhibition using VE-281 reduced MCF7 cell growth (Figure 3A in [133]), and NU6027 inhibition enhanced chemotherapeutic cytotoxicity to MCF7 cells (Figure 3 in [134]). Other ATR inhibitors, schisandrin B [135,136,137] and KU60019 [138,139], presented promising anti-tumor properties in vitro and partly in vivo in luminal A breast cancer models, however, they have a limited clinical use due to a poor bioavailability. Newer ATR inhibitors with better pharmacological properties (such as M6620, AZD6738 and BAY1895344) [140] have not been tested for luminal A breast cancer yet.

In conclusion, based on results of our GSEA analysis and knowledge from clinical trials we suggest the highest potential of alisertib and mesupron for CDK2 and PLAU-targeted therapy of lymph node negative luminal A breast cancer patients with high risk of distant metastasis.

### 3.6. Regulatory Mechanisms of T Lymphocyte Immune Response Play Role in Development of Distant Metastasis in Lymph Node Positive Luminal A Patients (N1: M1 vs. M0)

Comparison of tumors that develop distant metastases versus tumors that do not develop distant metastases during the follow-up period in the group of luminal A patients with positive lymph nodes (N1: M1 vs. M0) was associated with enrichment of genes belonging to TH1TH2 and CTLA4 pathways. These pathways participate in regulation of T lymphocyte differentiation and activation. CD40 and its ligand CD40L, signal molecules for immune and inflammatory responses [141] were enriched in TH1TH2 pathway. Tong et al. [142] detected CD40 expression in breast tumors including infiltrating ductal and lobular carcinomas and carcinomas in situ and showed weaker expression of CD40 in benign ductal epithelial tissues (Table 4 in [142]). CD40 and CD40L were associated with pathological grade and lymph node metastasis in breast cancer patients [72] and were related to immune response (Figures 1E,F and 2C in [143]). Gladue et al. reported that anti-CD40 antibody slowed the growth of the breast tumors down in SCID-beige mice model (Figure 4A,B in [144]). CD40L Inhibitor cyclosporin A [145] that already exerted potential for breast cancer therapy in vitro [146] represented in combination with docetaxel an effective treatment in patients with advanced breast cancer in phase II clinical trial [45]. Moreover, cyclosporin A significantly increased survival of patients with advanced non-small cell lung carcinoma [147]. CP-870,893, CD40 agonist antibody, induced antitumor activity for breast cancer in vivo [144] and is currently in phase I study in patients with pancreatic cancer and three other phase I trials in patients with advanced solid tumors [148]. CP-870,893 also reached clinical response in patients with metastatic melanoma in phase I trial [43].

CTLA4 pathway includes ITK, CTLA-4 and LCK biomarkers. ITK inhibitor ibrutinib inhibits luminal A breast cancer cells in vitro [149], represents safe treatment of solid tumors [150] and is currently being tested in a phase II clinical trial in Her2+ breast cancer patients (trial number: NCT03379428). Ibrutinib slowed down, in combination with anti–PD-L1 antibody, the growth of 4T1 tumors in BALB/c mice and reduced lung metastases (Figure 2 in [151]). ITK inhibition was reported to enhance T-cell anti-tumor immunity [73]. In term of CTLA4, its high expression in 130 breast tumors was significantly associated with shorter DFS (Figure 2 in [152]). Chen et al. demonstrated that antibody-based CTLA4 inhibition reduces proliferation and induces apoptosis of breast cancer cells (Figure 7B,D in [153]). Qu et al. [154] showed that CTLA4 antibody in combination with IL36 local overexpression inhibits lung metastasis growth in BALB/c mice model (Figure 3D,E, [154]). Anti-CTLA4 antibody tremelimumab in combination with exemestane supported tumor immunosuppression in ER+ breast cancer patients (phase I) [42]. Another CTLA4 inhibitor ipilimumab, an FDA-approved drug for treatment of melanoma, induced in combination with cryoablation antitumor activities of immune system in early stage breast cancer patients [44]. It is currently being tested, in combination with nivolumab, in two trials in breast cancer patients (Trial numbers: NCT01928394, NCT02833233). LCK is a regulatory factor in hypoxia-induced tumor progression and angiogenesis [155]. LCK expression was significantly higher in 81 metastatic breast tumors compared to 48 non-malignant and 10 normal breast tissue samples (Figure 1C in [156]). Moreover, LCK was detected in 30 early stage primary breast tumors and their lymph node metastases, but not in normal breast tissues (Figure 3 in [157]). LCK was found to play a crucial role in hypoxia/reoxygenation-induced migration of MCF7 and MDA-MB-231 cells (Figure 4 in [158]). Dasatinib and (–)-Liriopein B, Src inhibitors mentioned previously, inhibited LCK in breast cancer cells as well [107,108,112].

Based on our GSEA results and clinical trials we presume the highest potential of cyclosporin A and tremelimumab for targeted therapy of lymph node positive luminal A breast cancer patients with high risk of distant metastasis.

## 4. Materials and Methods

### 4.1. MMCI_706 Patient Cohort

706 breast cancer patients initially diagnosed in MMCI, Brno, Czech Republic between 2004 and 2007 were retrospectively enrolled in the study. Inclusion criteria were: availability of medical records for review, at least 5-year follow up. The key clinicopathological variables (age, tumor size, estrogen receptor, progesterone receptor and Her2 status, Ki-67 percentage of stained cells, tumor grade, lymph node status at the time of diagnosis, distant relapse) are available as Table S7. Exclusion criteria were as follows: cancer duplicity, metastatic disease at the time of diagnosis, neoadjuvant treatment, local relapse not accompanied by distant relapse during the follow-up period. Node positivity was considered as metastasis in one or more regional lymph nodes. Informed consent confirming the availability of redundant tissue samples for research use was obtained from each participating subject. MMCI_706 patient characteristics is summarized in Table 4.

This study was approved by Ethics committee of Masaryk Memorial Cancer Institute (2016/621/MOU, JID: MOU 107 995). All patients involved in this study gave permission to publish their anonymized clinical information.

### 4.2. Classification of MMCI_706 Patients into Molecular Subtypes

The estrogen receptor and progesterone receptor status were examined by immunohistochemistry (IHC), using antibodies provided by Lab Vision (SP1 resp. SP2 monoclonal rabbit antibody, Lab Vision Thermo Fisher Scientific, Fremont, CA, USA). ER and PgR status were considered positive if >1% of cells were stained in cell nuclei and was considered negative in all other cases. The expression of Her2 protein was determined by Dako Herceptest (Dako, Sweden) and scored on a qualitative scale from 0 to 3+ according to Dako manual and American Society of Clinical Oncology/College of American Pathologists guideline recommendations for human epidermal growth factor receptor 2 testing in breast cancer. HER2 gene status was evaluated by fluorescence in situ hybridization (FISH) method using Abbott PathVysion HER2 kit (Abbott Laboratories, Chicago, IL, USA). HER2 gene status was considered as positive (FISH amplified) in case where a HER2 gene/centromer of chromosome 17 ratio was higher than 2.2 or if the number of HER2 gene copies was higher than 6 per nucleus as measured by FISH. All Her2 positive tumors were IHC 3+ and/or FISH-positive [159]. Patients were classified into four molecular subtypes based on IHC profile according to Maisonneuve et al. [160] and St. Gallen 2013 consensus [161]. Luminal A patients were defined as ER+ and Her2- with low Ki-67 expression (<14%) or with intermediate Ki-67 expression (14% to 19%) and high PgR levels (≥20%). Luminal B patients were classified as ER+ and Her2- with intermediate Ki-67 expression (14% to 19%) and low PgR levels (<20%) or with high Ki-67 expression (≥20%) or ER+ and Her2+ with any Ki-67 and any PgR. Her2+ (non-luminal) patients showed over-expression or amplification of Her2, lacking ER and PgR expression. Basal/triple negative patients were ER, PgR and Her2 negative.

### 4.3. Publicly Available Microarray Dataset

Publicly available gene expression dataset SUPERTAM_HGU133A was downloaded from [32,162]. This dataset consists from MDA5, TAM, VDX and VDX3 datasets (all platform Affymetrix Human Genome U133A, 856 samples in total) originally deposited in Gene Expression Omnibus (GEO) database under the following IDs: GEO: GSE17705 (MDA5), GEO: GSE6532/GSE9195 (TAM), GEO: GSE2034/GSE5327 (VDX) and GEO: GSE12093 (VDX3) processed as previously described [32], please see Figure S8 for the diagram excluding a potential batch effect in SUPERTAM_HGU133A dataset. The most variable probeset per gene based on interquantile range (IQR) was selected. Samples with available lymph node status and documented distant relapse (836 samples in total) were classified into four molecular breast cancer subtypes using a SCMOD2 classification model [163], as it showed higher robustness [32] and better correlation [164] with St. Gallen 2011 classification [165] than commonly used PAM50. This resulted in 341 luminal A, 281 luminal B, 71 Her2+ and 143 basal patient samples. All calculations were performed in R 3.4.1 [166] using limma 3.32.2 package, SCMOD2 classification was performed using genefu package from Bioconductor [167]. Association between gene expression and local or distant metastases was assessed by moderated t-statistics (method implemented in the limma package) on the set of 13,091 genes. The most variable probesets per gene was selected from the original 22,283 probesets. *p*-values were adjusted for multiple hypothesis testing by Benjamini-Hochberg FDR correction.

### 4.4. Gene set Enrichment Analysis (GSEA)

To find the most involved pathways in the metastasis associated processes, we used javaGSEA 4.0.3 desktop application [168,169]. Student t-test was used for ranking the genes, minimal size of small sets for exclusion was set to 10, 1000 permutations were used, and default settings were applied for other parameters. Enrichment analysis applied pathways information from BIOCARTA database. Enrichment score (ES) was calculated for each gene set. Pathways were considered significant (i) if nominal *p*-value was below 0.05 and (ii) the pathways were enriched in lymph node positive or distant metastasis positive phenotype.

The processed data used for GSEA analysis and its raw outputs are available in Mendeley Data as dataset “SUPERTAM_HGU133A Gene Set Enrichment Analysis (GSEA) in term of lymph node and distant metastasis” [170].

### 4.5. Statistical Evaluation of Distant Metastatic Risk in Association with Nodal Metastasis

Association between risk of distant relapse and lymph node status was assessed in MMCI_706 and SUPERTAM_HGU133A patients for each breast cancer subtype. For this purpose, logistic regression (calculated in Microsoft Excel) was used with distant metastasis as the dependent variable and lymph node status as independent variable, Chi-Square Statistics was calculated in GraphPad Prism 8.4.3.

## 5. Conclusions

We observed that pathways associated with lymph node and distant metastasis are different and dependent on molecular subtype of the tumor. We identified panels of inhibitors based on GSEA that have potential to improve the outcome of luminal A breast cancer patients who (i) are lymph node positive, (ii) who are lymph node negative with higher risk of metastasis development, and (iii) who are lymph node positive with high risk of metastasis development. We hope that further clinical trials have potential to translate current knowledge from the laboratory to the improved treatment of breast cancer patients.

## Figures and Tables

**Table 1:** Increase of distant metastasis risk dependent on lymph node positivity in patients of four molecular subtypes from MMCI_706 study cohort and SUPERTAM_HGU133A dataset using logistic regression.

Tumor Molecular Subtype	MMCI_706				SUPERTAM_HGU133A			
	*n*	OR	95% CI	*p*-Value	*n*	OR	95% CI	*p*-Value
Luminal A	381	5.062	1.973–12.989	0.000	341	2.401	1.316–4.380	0.004
Luminal B	218	2.422	1.151–5.096	0.018	281	1.386	0.787–2.442	0.258
Her2+	32	3.462	0.32–37.475	0.285	71	5.375	1.421–20.332	0.008
Basal	75	4.400	1.479–13.091	0.006	143	0.299	0.037–2.448	0.416
All patients	706	3.634	2.228–5.928	0.000	836	1.739	1.207–2.505	0.003
Luminal A + luminal B	599	3.536	1.994–6.271	0.000	622	1.762	1.176–2.641	0.006
Her2+ + basal	107	3.948	1.492–10.450	0.004	214	1.864	0.752–4.619	0.174

CI, confidence interval; OR, odds ratio.

**Table 2:** BIOCARTA pathways (NOM *p*-value < 0.05) significantly enriched in luminal A primary tumors in SUPERTAM_HGU133A dataset including core enriched genes and their inhibitors.

Pathway Name	Core Enriched Genes	Inhibitors
SUPERTAM_HGU133A Luminal A—N1 vs. N0		
RANKL	FOS, MAPK8, TNFSF11, RELA, TRAF6, FOSL1, IFNAR1, TNFRSF11A	denosumab, curcumin, parthenolide, BAY-11-7082, DHMEQ
CELL2CELL	ACTN1, PECAM1, CTNNA3, SRC, ACTN2, CSK, CTNNA2	dasatinib, PP2, nobiletin, (-)-Liriopein B
SUPERTAM_HGU133A Luminal A—N1: M1 vs. M0		
TH1TH2	CD86, IL2RA, CD40, CD40LG, IFNG, IL12RB1, CD28, HLA-DRB1	cyclosporin A, CP-870,893
CTLA4	CD86, LCK, CD3D, CD80, ITK, CD3E, GRB2, ICOSLG, TRA@, CTLA4, CD28, HLA-DRB1, CD247	tremelimumab, ipilimumab, ibrutinib, dasatinib, (-)-Liriopein B
SUPERTAM_HGU133A Luminal A—N0: M1 vs. M0		
MCM	MCM4, MCM2, CDC6, MCM6, CDK2, CDKN1B	alisertib, cepharantine, roscovitine, norcantharidin, lycopene, troglitazone, SNS-032, trichostatin A, NU2058, NU6102, SU9516, furanodiene, MHY412, retinoic acid, AZD5438, ICEC-0782, euphol, tehranolide, gallic acid, pentagalloylglucose, 15,16-dihydrotanshinone I, hesperetin
FIBRINOLYSIS	PLAT, PLAU, F13A1, SERPINB2, F2R	mesupron, nimbolide
ATRBRCA	MRE11A, BRCA1, BRCA2, ATR, RAD9A, FANCG, RAD51, FANCF, HUS1	schisandrin B, NU6027, VE-821, KU60019

N0: M1 vs. M0, distant metastasis positive vs. negative tumors; all lymph node negative; N1: M1 vs. M0; distant metastasis positive vs. negative tumors; all lymph node positive; N1 vs. N0, lymph node positive vs. negative tumors.

**Table 3:** Overview of clinical trials of selected inhibitors.

Inhibitor Name	Condition	Pathway	Target	Disease	Outcomes of Clinical Trials	Ref.
Denosumab	N1 vs. N0	RANKL	RELA	**Breast cancer**	Reduced bone turnover and bone events (phase II)	[34]
					Improved DFS in ER+ patients due to reduced occurrence of clinical fractures (phase III)	[35]
Dasatinib	N1 vs. N0	CELL2CELL	Src	**Breast cancer**	Showed clinical activity with paclitaxel in metastatic patients, but with slow accrual (phase II)	[36]
				**Breast cancer**	Limited single-agent activity in ER+ patients (phase II)	[37]
				**Breast cancer**	Dasatinib + zoledronic acid was well tolerated with responses in ER+ patients (phase II)	[38]
				**Breast cancer**	Dasatinib + trastuzumab prolonged progression-free survival in Her2+ breast cancer patients (phase II)	[39]
Curcumin	N1 vs. N0	RANKL	RELA	Solid cancer	Well tolerated in patients with local advanced and metastatic cancer (phase I)	[40]
				**Breast cancer**	In combination with hydroxytyrosol and omega-3 fatty acids reduced inflammation and pain	[41]
Tremelimumab	N1: M1 vs. M0	CTLA4	CTLA4	**Breast cancer**	Tremelimumab + exemestane maintained a stable disease in 42% patients (phase I)	[42]
				Melanoma	Tremelimumab + CP-870,893 reached overall response rate in 27.2% patients (phase I)	[43]
Ipilimumab	N1: M1 vs. M0	CTLA4	CTLA4	**Breast cancer**	Safe in early stage breast cancer patients with potential to induce immune antitumor activities	[44]
Cyclosporin A	N1: M1 vs. M0	TH1TH2	CD40LG	**Breast cancer**	Cyclosporin A + docetaxel was an effective and safe treatment in patients with advanced disease (phase II)	[45]
				Lung cancer	Increased survival of patients (phase I/II)	[46]
CP-870,893	N1: M1 vs. M0	TH1TH2	CD40LG	Solid tumors	Well tolerated with observed antitumor activity (phase I)	[47]
				Pancreatic cancer	CP-870,893 + gemcitabine was well-tolerated and associated with antitumor activity	[48]
				Melanoma	CP-870,893 + tremelimumab reached overall response rate in 27.2% patients (phase I)	[43]
Ibrutinib	N1: M1 vs. M0	CTLA4	ITK	**Breast cancer**	Clinical trial with Her2+ patients is in process (phase II)	NCT03379428
Alisertib	N0: M1 vs. M0	MCM	CDK2	**Breast cancer**	Alisertib + paclitaxel showed promising antitumor activity (phase II)	[49]
				**Breast cancer**	Alisertib + fulvestrant showed antitumor activity in metastatic, endocrine-resistant, ER+ patients (phase I)	[50]
Cepharantine	N0: M1 vs. M0	MCM	CDK2	**Breast cancer**	CEP showed an efficacy on preventing leukocytopenia induced by chemotherapy in breast cancer patients	[51]
				**Breast cancer**	CEP prevented bone marrow suppression induced by adjuvant chemotherapy in breast cancer patients	[52]
Roscovitine	N0: M1 vs. M0	MCM	CDK2	**Breast cancer**	Roscovitine + capecitabine in metastatic patients, no results available (phase II)	[53]
				Solid tumors	Roscovitine + sapacitabine show antitumor activity in metastatic patients with BRCA mutations (phase I)	[54]
				Nasopharyngeal cancer	Roscovitine was effective in reducing cervical lymph node size and maintaining stable disease	[55]
Norcantharidin	N0: M1 vs. M0	MCM	CDK2	Hepatic cancer	Clinically used to treat liver cancer in China	[56]
Lycopene	N0: M1 vs. M0	MCM	CDK2	Prostate cancer	Reduced disease progression with decreased serum prostate-specific antigen concentrations	[57]
Troglitazone	N0: M1 vs. M0	MCM	CDK2	Prostate cancer	Increased incidence of prolonged stabilization of prostate-specific antigen	[58]
SNS-032	N0: M1 vs. M0	MCM	CDK2	Solid tumors	SNS-032 was well tolerated (phase I)	[59]
Mesupron	N0: M1 vs. M0	FIBRINOLYSIS	PLAU	**Breast cancer**	Mesupron + capecitabine improved PFS in Her2- metastatic patients (phase II)	[60]
				Pancreatic cancer	Mesupron + gemcitabine increased patient survival (phase II)	[61]

BRCA, breast cancer susceptibility protein; CEP, cepharantine; DFS, disease-free survival; ER, estrogen receptor; Her2, human epidermal growth factor receptor 2; N0: M1 vs. M0, distant metastasis positive vs. negative tumors; all lymph node negative; N1: M1 vs. M0; distant metastasis positive vs. negative tumors; all lymph node positive; N1 vs. N0, lymph node positive vs. negative tumors; PFS, progression-free survival. Breast cancer clinical trials are highlighted in **bold**.

**Table 4:** Patient characteristics in MMCI_706 set of patients.

MMCI_706	All	Luminal A	Luminal B	Her2+	Basal
	(*n* = 706)	(*n* = 381)	(*n* = 218)	(*n* = 32)	(*n* = 75)
Age (years)					
median	57	59	55	53.5	53
<60	414 (58.6%)	181 (47.5%)	139 (63.8%)	23 (71.9%)	52 (69.3%)
≥60	292 (41.4%)	200 (52.5%)	79 (36.2%)	9 (28.1%)	23 (30.7%)
pT					
T1	431 (61.0%)	255 (66.9%)	120 (55.0%)	21 (65.6%)	35 (46.7%)
T2	237 (33.6%)	107 (28.1%)	85 (39.0%)	10 (31.3%)	35 (46.7%)
T3–4	36 (5.1%)	19 (5.0%)	12 (5.5%)	-	5 (6.6%)
NA	2 (0.3%)	-	1 (0.5%)	1 (3.1%)	-
Grade					
G1	238 (33.7%)	207 (54.3%)	29 (13.3%)	-	2 (2.7%)
G2	259 (36.7%)	151 (39.7%)	101 (46.3%)	3 (9.4%)	4 (5.3%)
G3	203 (28.8%)	21 (5.5%)	87 (39.9%)	26 (81.2%)	69 (92.0%)
NA	6 (0.8%)	2 (0.5%)	1 (0.5%)	3 (9.4%)	-
ER					
negative	107 (15.2%)	-	-	32 (100%)	75 (100%)
positive	599 (84.8%)	381 (100%)	218 (100%)	-	-
HER2					
negative	628 (88.9%)	381 (100%)	46 (21.1%)	-	75 (100%)
positive	78 (11.1%)	-	172 (78.9%)	32 (100%)	-
Nodes					
negative	388 (55.0%)	225 (59.1%)	108 (49.5%)	16 (50.0%)	39 (52.0%)
positive	318 (45.0%)	156 (40.9%)	110 (50.5%)	16 (50.0%)	36 (48.0%)
Distant metastasis					
negative	617 (87.4%)	356 (93.4%)	180 (82.6%)	28 (87.5%)	53 (70.7%)
positive	89 (12.6%)	25 (6.6%)	38 (17.4%)	4 (12.5%)	22 (29.3%)
Adjuvant chemotherapy					
no	335 (47.5%)	246 (64.6%)	73 (33.5%)	6 (18.8%)	10 (13.3%)
yes	371 (52.5%)	135 (35.4%)	145 (66.5%)	26 (81.2%)	65 (86.7%)

ER, estrogen receptor; Her2, human epidermal growth factor receptor 2; pT, tumor size category.

## Supplementary Materials

The following are available online at https://www.mdpi.com/2072-6694/12/9/2638/s1, Table S1: Overview of regional lymph node metastasis distribution in tumors of different breast cancer subtypes, Table S2: Overview of distant metastasis distribution in tumors of different breast cancer subtypes, Table S3: Association between lymph node metastases and distant metastases in patients from MMCI_706 study cohort and SUPERTAM_HGU133A dataset, Table S4: Increase of distant metastasis risk depending on lymph node positivity in patients of four molecular subtypes from MMCI_706 cohort with and without chemotherapy in adjuvant treatment using logistic regression, Table S5: Differential expression (at protein and transcript level) of NF-κB associated proteins RELA, PDLIM2, RNF25 and TRAF3IP2 in lymph node positive vs. lymph node negative, grade 1 luminal A breast tumors (G1: N1–2/N0), Table S6: Results of Gene Set Enrichment Analysis (GSEA) of SUPERTAM_HGU133A dataset: GSEA enriched pathways and genes; Table S7: MMCI_706 patient characteristics including the key clinicopathological variables (age, tumor size, estrogen receptor, progesterone receptor and Her2 status, Ki-67 percentage of stained cells, tumor grade, lymph node status at the time of diagnosis, distant relapse), Figure S1: Hierarchical clustering of top 500 differentially expressed genes within luminal A subtype for various combinations of lymph node and distant metastasis status, Figures S2–S7: Volcano plots for various combinations of lymph node and distant metastasis status in luminal A patients, Figure S8: Boxplot diagram showing total log2 gene expression intensities for all 856 patient samples in the SUPERTAM_HGU133A dataset to exclude a potential batch effect.
